# Impact of *CYP1A*, *CYP2C19*, *CYP2D6*, *CYP3A4*, *CYP3A5*, and *NFIB* genotypes on clozapine serum concentration in smokers and nonsmokers

**DOI:** 10.1177/20451253251377183

**Published:** 2025-10-04

**Authors:** Hasan Çağın Lenk, Line Skute Bråten, Ole A. Andreassen, Espen Molden

**Affiliations:** Center for Psychopharmacology, Diakonhjemmet Hospital, Oslo, Norway; Centre for Precision Psychiatry, Faculty of Medicine, University of Oslo, Oslo, Norway; Center for Psychopharmacology, Diakonhjemmet Hospital, Oslo, Norway; Centre for Precision Psychiatry, Faculty of Medicine, University of Oslo, Oslo, Norway; Section for Precision Psychiatry, Oslo University Hospital, Oslo, Norway; Center for Psychopharmacology, Diakonhjemmet Hospital, Forskningsveien 13, PO Box 85 Vinderen, Oslo 0319, Norway; Department of Pharmacy, Section for Pharmacology and Pharmaceutical Biosciences, University of Oslo, Oslo, Norway

**Keywords:** personalized treatment, pharmacogenetics, pharmacokinetics, precision psychiatry, treatment resistant schizophrenia

## Abstract

**Background:**

Clozapine is the most effective drug for schizophrenia and is the only drug indicated for use in patients with treatment resistance. The therapeutic range of clozapine is narrow with extensive interindividual differences in serum levels at similar dosing, mainly due to variability in hepatic metabolism mediated by several cytochrome P450 (CYP) enzymes. Tobacco smoking is the most important environmental factor determining clozapine metabolism, while the effect of pharmacogenetic variability is unclear.

**Objectives:**

To investigate the impact of *CYP1A, CYP2C19, CYP2D6, CYP3A4, CYP3A5*, and *NFIB* alleles on clozapine levels stratified by smoking status in a large patient population.

**Design:**

This is a retrospective naturalistic/observational study.

**Methods:**

The study population was included from the therapeutic drug monitoring/pharmacogenetics service at the Center for Psychopharmacology, Diakonhjemmet Hospital, Oslo, Norway, during January 2005–November 2024. We assessed the influence of *CYP1A* rs247229 *T*, CYP1A2*1F, *CYP2C19*, *CYP2D6*, CYP3A4*22 and CYP3A5*3, and *NFIB* rs28379954 *C* genetic variants on clozapine dose-adjusted serum concentrations (CD) in both smokers and nonsmokers.

**Results:**

The study comprised 663 participants (55% smokers). *CYP1A T* variant was significantly associated with reduced clozapine serum levels, compared to *CYP1A CC* genotype, both among smokers (–15%, *p* = 0.010) and nonsmokers (–16%; *p* = 0.011). Moreover, among smokers, participants with *NFIB C* variant had 40% reduced clozapine CD compared to participants with *NFIB TT* (*p* < 0.001), whereas carriers of the CYP3A5*1/*1 genotype exhibited a 37% lower clozapine CD compared to CYP3A5*3/*3 carriers (*p* = 0.024) among nonsmokers. CYP1A2*1F, *CYP2C19*, *CYP2D6*, and CYP3A4*22 variants did not have any significant impact on clozapine CD, regardless of smoking habits.

**Conclusion:**

The *CYP1A T*, *NFIB C*, and CYP3A5*1 alleles have significant impact on clozapine serum levels. Incorporating genotype information for these variants, together with patient smoking status, would improve algorithms for precision dosing of clozapine.

## Introduction

Schizophrenia is a chronic and debilitating psychotic disorder with a 15-year shorter life expectancy and high risk of physical comorbidities such as cardiovascular disease.^1,2^ Treatment with antipsychotic medications has been shown to be effective in improving symptoms^3,4^ and reducing mortality.^5^ However, treatment response of antipsychotics shows large interindividual variability and 20%–30% of patients experience persistent symptoms after trying two or more different antipsychotics in adequate doses, which is defined as treatment resistant schizophrenia (TRS).^6^

Clozapine is a second-generation antipsychotic, and the only drug indicated for use in TRS. Compared to other antipsychotics, clozapine has been shown to have greater efficacy in ameliorating symptoms^7,8^; however, its use is restricted for management of TRS due to risk of potentially life-threatening granulocyte toxicity, which necessitates mandatory hematological follow-up.^9^ Another major challenge is that clozapine exhibits high interindividual variation in serum concentrations, which is associated with clinical response and tolerability of clozapine treatment.^10^ While 350 ng/ml is defined as the lower boundary to achieve response,^6,10^ 600 ng/ml is the recommended upper threshold^11,12^ as increasing concentrations are shown to increase the risk of severe dose-dependent side effects such as tonic-clonic seizures.^13,14^ Therefore, therapeutic drug monitoring (TDM) of clozapine is recommended to guide dosing to ensure optimal clinical response and reduced risk of concentration-dependent side effects.^12^

The extensive pharmacokinetic variability of clozapine is mainly due to individual variability in complex hepatic metabolism mediated by several cytochrome P450 (CYP) enzymes. The major phase I metabolic pathways of clozapine comprise two oxidative reactions. Most attention has been paid to the *N*-demethylation pathway, both because the metabolite has been associated with treatment effect and tolerability^15^ and quantitatively most important pathway. Studies suggest that *N*-demethylation of clozapine is catalyzed mainly by CYP1A2 and CYP3A4, whereas CYP2C19 and CYP2D6 are referred to play only minor roles.^16,17^ Less research has been performed to reveal enzyme(s) responsible for the second oxidative pathway, that is, *N*-oxidation. However, in vitro studies indicate that CYP3A4 is the major enzyme mediating *N*-oxidation of clozapine, with CYP1A2 playing a secondary role.^16,17^ Recent studies have linked *N*-oxide clozapine to risk of cardiovascular toxicity,^18,19^ which implies that increased attention should be paid to this metabolic reaction in relation clinical outcome of clozapine treatment.

While metabolism via CYP2C19 and CYP2D6 is highly dependent genetic on variability,^20^ activities of CYP1A2 and CYP3A4 are less determined by genetic factors.^21,22^ For clozapine, tobacco smoking status is by far the most important environmental factor determining its metabolism and leads up to 40% reduction in serum levels by inducing CYP1A2 expression.^20,23^ As a consequence, performing reliable pharmacogenetic studies with clozapine require information on smoking habits. This point was clearly shown in a recent study where an effect of the *NFIB* rs28379954 *T>C* (*NFIB C*) variant on clozapine serum level was only evident after correcting for smoking status.^24^ However, so far, pharmacogenetic studies on clozapine have considered smoking status to a limited extent due to small sample size which hindered the stratification by smoking status with sufficient statistical power. This may be a reason why inconsistent results have been reported in different studies.^25–29^ In real-life studies, it is also essential with knowledge on drug–drug interactions that impact the metabolic capacity of CYP enzymes involved in clozapine metabolism to precisely predict genotype effects on clozapine serum levels. Indeed, compared to population-based models, personalized dose prediction algorithms of clozapine perform significantly better when genetic activity scores and interacting comedications are included in the models.^30^

Several pharmacogenetic variants have been reported to be associated with clozapine serum levels; however, current evidence is contradictory for clinical usefulness of these variants. Current international guidelines on clozapine titration^31^ and Dutch Pharmacogenetics Working Group (DPWG) guideline for gene–antipsychotics interaction^32^ indicate that genotyping for *CYP1A2* and other genes involved in clozapine metabolism do not provide useful information for personalized dosing. In the clozapine package insert, initially approved by the Food and Drug administration (FDA) in the late 1980s, it is stated that CYP2D6 poor metabolizers (PMs) may have increased clozapine serum concentration and lowering clozapine dose may be required.^33^ However, later studies reported limited or no effect of *CYP2D6* genotype clozapine levels.^26,34^ Based on findings from genome-wide investigations on clozapine serum levels,^24,35^ we have recently reported significantly reduced clozapine serum levels associated with *NFIB C* and *CYP1A* rs2472297 *T* (*CYP1A T*) variant alleles, especially among smokers.^36^ Furthermore, the promotor variant CYP1A2*1F has been associated with lower levels of clozapine, but the results in different studies are inconsistent.^37,38^

Discovery of genetic variants determining variability in CYP3A4 metabolism is limited, except from CYP3A4*22 (intron 6 *C>T*), which has a relatively low allele frequency in most populations, for example, 5.0% in Europeans, 2.6% in the admixed American population and <1% for the Asian and African population.^39^ However, for CYP3A5, which is a CYP3A isoform sharing substrate specificity with CYP3A4, enzyme activity shows extensive dependency on genetics. Pharmacogenetics of *CYP3A5* differ from other *CYP* genes, as major parts of populations express no enzyme (CYP3A5*3), whereas the minorities carry the CYP3A5*1 allele defined as wild type (*wt*). Patients carrying the functional variant, that is, CYP3A5*1, ranges from 10%–15% in Caucasians to 40%–50% in Black Africans/African Americans.^39^ Both CYP3A4 activity and *CYP3A5* genotype have been associated with clozapine serum levels, but the findings are conflicting.^25,28,40^

Since historical evidence on the impact of genetic variation in *CYP-* genes on clozapine serum levels is inconsistent and studies conducted include small population sizes with limited access to smoking status, we studied the quantitative effects of the genetic variants in the *CYP1A*, *CYP2C19*, *CYP2D6*, *CYP3A4*, *CYP3A5*, and *NFIB* on clozapine serum levels in a Norwegian population of 663 participants with known smoking status and longitudinal TDM profiles of clozapine.

## Methods

The reporting of this study conforms to the Strengthening the Reporting of Observational Studies in Epidemiology (STROBE) statement (Supplemental Material).^41^

### Study population

The study population was included retrospectively from the routine TDM/pharmacogenetics service at the Center for Psychopharmacology, Diakonhjemmet Hospital, Oslo, Norway, during January 2005 to November 2024. TDM and *CYP* genotyping are reimbursed in Norway and utilized as a tool in psychiatric clinical practice for personalizing treatment to patients’ needs including optimized dosing to attain therapeutic levels, determination of metabolizer status for certain medications, and follow-up of patients to assess treatment stability over time.

Current study population comprised 60% of participants (*n* = 395) from our previous study,^36^ while the remaining *n* = 268 participants were newly included. Adult participants with a history of detectable clozapine serum concentration and available genotype information for at least one of the genes investigated (i.e., *CYP1A, CYP2C19*, *CYP2D6*, *CYP3A4*, *CYP3A5*) were included in the study. In addition to assessing these *CYP* genotypes, a comparison between carriers of the *NFIB C* versus *T* alleles was performed. *CYP2C19* and *CYP2D6* genotypes were retrieved from the laboratory database, where they had been previously performed as part of the routine pharmacogenetic service at the Center for Psychopharmacology. In contrast, genotyping of *CYP1A*, *CYP3A4*, *CYP3A5*, and *NFIB* variants was performed on available residual biobanked blood samples for research purposes. Overall, as genotyping was based on sample availability and historical testing, the number of patients with data for each genetic variant varied, and individuals included in the study did not necessarily have genotype information for all investigated loci. Clozapine measurements included in the study were assumed to be at steady state as it is one of the requirements for clozapine TDM stated in the requisition forms and the study commonly included participants with longitudinal TDM profiles with several clozapine measurements over time. Study population consisted of Norwegian inhabitants and was assumed to be mostly of Caucasian ancestry based on the ethnic composition of the Norwegian population (ethnicity not confirmed in the study).

Exclusion criteria were (1) prescribed dose of clozapine outside the clinical range (100–1000 mg/day) where non-linear clozapine pharmacokinetics are likely, (2) time between blood sampling and last dose intake (withdrawal time) outside 10–30 h, (3) participants older than 65 years at the time of blood sampling, and (4) lack of consistent information on smoking status (yes/no written on the requisition form). Additionally, participants were excluded from the study if they had a history of detectable levels of comedications that may interfere with clozapine metabolism including (1) CYP inducers (carbamazepine, phenobarbital, and phenytoin), (2) CYP2D6 inhibitors (bupropion, fluoxetine, and paroxetine), (3) CYP1A2/3A4 inhibitor fluvoxamine, (4) CYP2C19 inhibitor omeprazole, and (5) valproate due to a special interaction with clozapine metabolism.^42^ Information on age, sex, prescribed daily dosage, comedications, withdrawal time, and smoking habits were obtained from the clozapine TDM requisition forms. TDM records lack clinical information; therefore, we were unable to evaluate therapeutic response scales as part of the current study.

### TDM analysis of clozapine serum concentration

Serum concentration analysis of clozapine in TDM practices are performed using liquid chromatography with mass spectrometry detection, which has been described in detail elsewhere.^43^ There was a change in the methods for serum concentration analysis during the patient inclusion period due to a renewal of instrumentation; however, all the methods were cross-validated and accredited for routine TDM analysis.

The latest method, ultra-high-performance LC (UHPLC) system coupled with a high-resolution MS (HRMS) Orbitrap^®^ (Thermo Fisher Scientific, Waltham, MA, USA) detector has been applied since 2018 and provided us with nonselective full-scan TDM data, which makes it possible to retrospectively reprocess for molecular targets without reanalyzing the samples. We utilized this to reprocess the latest occurring clozapine samples for each patient (*n* = 389), when available, for additional identification of relevant comedications for exclusion. These medications (with available timeframes) were: carbamazepine, bupropion, fluoxetine, paroxetine, and fluvoxamine (after 2018) and omeprazole and valproate (after 2019).

### Genetic analyses

*CYP2C19* and *CYP2D6* genotypes were analyzed using certified real time polymerase chain reaction (PCR) with TaqMan assays and copy number analyses. The *CYP2C19* genotyping assay included the nonfunctional alleles CYP2C19*2 (rs4244285), CYP2C19*3 (rs4986893), and CYP2C19*4 (rs28399504) and the increased expression allele CYP2C19*17 (rs12248560). Analyses of *CYP2D6* included the nonfunctional alleles CYP2D6*3 (rs35742686), CYP2D6*4 (rs3892097), CYP2D6*5 (whole gene deletion), CYP2D6*6 (rs5030655), the decreased function variants CYP2D6*9 (rs5030656), CYP2D6*10 (rs1065852), CYP2D6*41 (rs28371725), and duplicated functional *CYP2D6* alleles. Lack of any of the analyzed *CYP2C19* or *CYP2D6* variant alleles was interpreted as the wild-type (*1) allele for the corresponding gene. Classifications of genotype-predicted metabolizer phenotype of CYP2C19 and CYP2D6 were based on DPWG recommendations^44^ and a previous study,^45^ respectively, as shown in Table 1.

**Table 1.** Genotype-phenotype translations of CYP2C19 and CYP2D6.

Gene	Genotype	Genotype-predicted metabolizer phenotype
*CYP2C19*	*Nonf/Nonf*	Poor metabolizer
	*1/Nonf, Nonf/*17	Intermediate metabolizer
	*1/*1, *1/*17	Normal metabolizer
	*17/*17	Ultrarapid metabolizer
*CYP2D6*	*Nonf/Nonf*	Poor metabolizer
	*Nonf/Decr, Decr/Decr*	Intermediate metabolizer
	*1/Nonf, *1/Decr	Intermediate metabolizer+
	*1/*1	Normal metabolizer
	*1/*1× *N*	Ultrarapid metabolizer

Nonf: *2,*3, *4 for *CYP2C19* and *3, *4, *5, *6 for CYP2D6. Decr: *9, *10, *41 for *CYP2D6*.

Pharmacogenetic analyses of *CYP1A*, *CYP3A4*, *CYP3A5*, and *NFIB* variants were accomplished using TaqMan PCR assays; *CYP1A* rs2472297 *C>T* (C__11773054_10), CYP1A2*1F (rs762551; C__8881221_40), CYP3A4*22 (rs35599367; C__59013445_10), CYP3A5*3 (rs776746; C__26201809_30), and *NFIB* rs28379954 *T>C* (C__59359617_10).

### Outcomes

The primary outcome was to assess the influence of *CYP-* and *NFIB* genetic variants on dose-adjusted serum concentrations (CD), which was analyzed separately for smokers and nonsmokers. In TDM research, CD (nmol/L/mg/day) is commonly used to evaluate pharmacokinetic variability of a drug and reflects overall drug clearance. It is calculated by dividing the measured serum concentration of clozapine (nmol/L) by the prescribed daily dose (mg/day).

### Statistical analysis

To fully utilize all clozapine TDM measurements of clozapine during the study period, multivariate linear mixed-effects models with random intercepts and restricted maximum likelihood estimation were employed. All outcome variables in the mixed models were log-transformed (natural logarithm scale) prior to analysis; however, the estimated marginal means and 95% confidence intervals (CIs) were back-transformed to the linear scale for result presentation. To assess the effect of genetic variants on clozapine CD and absolute concentration, the study population was stratified based on smoking status. Fixed-effect factors included individual genotypes, age, sex, and withdrawal time, while anonymized patient identification numbers were included as random effects. The total daily prescribed dose was included as an additional covariate in models analyzing absolute clozapine serum concentrations. There was also a mixed-model analysis of CD where we included all the genotypes with similar covariates.

All statistical analyses were conducted using R (version 4.4.3).^46^ Linear mixed-effects models were performed using the R packages lme4 (version 1.1.37) and lmerTest (version 3.1.3).^47^ Marginal means derived from the mixed models, and subsequent group comparisons were calculated with the emmeans (version 1.11)^48^ and parameters (version 0.24.2)^49^ packages. As the design of the current study was hypothesis-driven and confirmatory based on previous literature (not genome-wide or exploratory), the correction for multiple gene testing was not performed and nominal *p*-values were reported. Statistical significance was set at a *p*-value smaller than 0.05.

## Results

The study population comprised 663 participants with a total of 8764 clozapine TDM measurements during the study period. The demographic and TDM-related characteristics of the participants are presented in Table 2. The median number of clozapine TDM measurements per subject was 6 (interquartile range (IQR): 2–16), covering a median observation period of 2.6 years (IQR: 0.1–8.1). Of the study subjects, 38% were female, and the mean age was 37.4 years (95% confidence interval (CI): 36.5–38.3). Across all TDM measurements, the estimated mean withdrawal time was 13.7 h (95% CI: 13.6–13.9). Additionally, 55% of the study population (*n* = 365; Table 2) were identified as smokers who were associated with 37% decreased clozapine CD compared to nonsmokers (fold change: 0.63; 95% CI: 0.58–0.69; *p* < 0.001). Overall, the allele frequencies of the various genetic variants in our study were comparable to those observed in European populations.

**Table 2.** Population and treatment characteristics of the study population.

Variable	Value
Number of subjects (samples), *n*	663 (8764)
Number of samples per subject; median (IQR)	6.0 (2.0, 16.0)
Duration of clozapine TDM per subject, years; median (IQR)	2.6 (0.1, 8.1)
Female, *n* (%)	254 (38)
Age, years; mean (95% CI)	37.4 (36.5, 38.3)
Withdrawal time, h; mean (95% CI)	13.7 (13.6, 13.9)
Smokers, *n* (%)	365 (55)

Withdrawal time is the time difference between the last clozapine intake and blood sampling for TDM.

CI, confidence interval; IQR, interquartile range; TDM, therapeutic drug monitoring.

Table 3 provides an overview of the estimated means and comparisons of quantitative effects of genetic variants on clozapine CD. Presence of the *CYP1A T* variant was associated with reductions of 15% (fold-change: 0.85; 95% CI: 0.76–0.96, *p* = 0.010; Table 3) and 16% (fold-change: 0.84; 95% CI: 0.73–0.96; *p* = 0.011) clozapine CD compared to wild-type genotype carriers among smokers and nonsmokers, respectively. In contrast, no significant changes in clozapine CD were observed among carriers of the CYP1A2*1F haplotype compared to homozygous major CYP1A2*1 carriers, either among smokers (*1/*1F vs *1/*1, *p* = 0.12; *1F/*1F vs *1/*1, *p* = 0.15; Table 3) or nonsmokers (*1/*1F vs *1/*1, *p* = 0.61; *1F/*1F vs *1/*1, *p* = 0.35; Table 3).

**Table 3.** Estimated means of dose-adjusted serum concentrations (nmol/L/mg/day; CD) of clozapine according to different *CYP-* and *NFIB* genotypes in smokers and nonsmokers.

Genotypes	Smokers				Nonsmokers			
	*n*, subjects (samples)	Estimated Mean (95%CI)	Fold change (95% CI)	*p* value	*n*, subjects (samples)	Estimated Mean (95%CI)	Fold change (95% CI)	*p* value
*CYP1A* rs2472297 *C>T*								
*CC*	180 (2795)	2.84 (2.62, 3.08)	—	—	143 (2132)	4.59 (4.21, 5.0)	—	—
*TC/TT*	127 (1825)	2.42 (2.21, 2.66)	**0.85 (0.76, 0.96)**	**0.010**	90 (1324)	3.84 (3.45, 4.27)	**0.84 (0.73, 0.96)**	**0.011**
*CYP1A2*								
*1/*1	28 (484)	3.08 (2.53, 3.75)	—	—	21 (414)	4.65 (3.71, 5.85)	—	—
*1/*1F	130 (1954)	2.60 (2.36, 2.85)	0.84 (0.68, 1.05)	0.12	104 (1494)	4.36 (3.94, 4.83)	0.94 (0.73, 1.2)	0.61
*1F/*1F	149 (2182)	2.63 (2.4, 2.87)	0.85 (0.69, 1.06)	0.15	108 (1548)	4.14 (3.75, 4.57)	0.89 (0.69, 1.14)	0.35
*CYP2C19*								
NM	223 (2801)	2.53 (2.34, 2.75)	—	—	188 (2146)	4.09 (3.79, 4.42)	—	—
PM	4 (96)	3.07 (1.82, 5.18)	1.21 (0.71, 2.06)	0.47	10 (91)	5.42 (3.85, 7.62)	1.32 (0.93, 1.88)	0.12
IM	87 (1271)	2.86 (2.54, 3.23)	1.13 (0.98, 1.3)	0.09	66 (938)	4.49 (3.94, 5.12)	1.10 (0.94, 1.28)	0.23
UM	7 (55)	3.31 (2.16, 5.06)	1.30 (0.85, 2.01)	0.23	5 (20)	4.80 (2.91, 7.93)	1.17 (0.71, 1.95)	0.53
*CYP2D6*								
NM	124 (1481)	2.81 (2.53, 3.12)	—	—	100 (954)	4.30 (3.86, 4.78)	—	—
PM	17 (224)	2.18 (1.66, 2.87)	0.78 (0.58, 1.04)	0.09	19 (213)	4.26 (3.32, 5.47)	0.99 (0.76, 1.3)	0.96
IM	17 (230)	2.63 (2.01, 3.44)	0.94 (0.7, 1.25)	0.65	18 (77)	4.12 (3.19, 5.33)	0.96 (0.73, 1.27)	0.77
IM+	121 (1628)	2.63 (2.37, 2.92)	0.94 (0.81, 1.08)	0.36	109 (1442)	4.36 (3.94, 4.82)	1.01 (0.88, 1.17)	0.85
UM	10 (161)	2.59 (1.82, 3.67)	0.92 (0.64, 1.32)	0.65	7 (50)	4.13 (2.73, 6.24)	0.96 (0.63, 1.47)	0.85
*CYP3A4*								
*1/*1	286 (4369)	2.68 (2.51, 2.86)	—	—	213 (3184)	4.25 (3.96, 4.56)	—	—
*1/*22	21 (251)	2.35 (1.87, 2.96)	0.88 (0.69, 1.11)	0.28	20 (272)	4.61 (3.65, 5.83)	1.08 (0.85, 1.39)	0.51
*CYP3A5*								
*3/*3	242 (3656)	2.71 (2.52, 2.90)	—	—	184 (2846)	4.27 (3.96, 4.6)	—	—
*1/*3	55 (776)	2.37 (2.05, 2.74)	0.87 (0.75, 1.02)	0.10	42 (574)	4.67 (3.99, 5.48)	1.10 (0.92, 1.3)	0.31
*1/*1	10 (188)	2.97 (2.16, 4.1)	1.1 (0.79, 1.52)	0.57	7 (36)	2.67 (1.79, 3.98)	**0.63 (0.42, 0.94)**	**0.024**
*NFIB* rs28379954 *T>C*								
*TT*	280 (4266)	2.76 (2.59, 2.95)	—	—	212 (3222)	4.32 (4.03, 4.64)	—	—
*CT/CC*	27 (354)	0.61 (0.49, 0.75)	**0.60 (0.49, 0.74)**	**<0.001**	21 (234)	3.90 (3.12, 4.88)	0.90 (0.71, 1.14)	0.39

Multivariate linear mixed-effects models were used to estimate the impact of pharmacogenetic variants separately among smokers and nonsmokers. All models included age, gender, and withdrawal time as covariates. Outcome variables in the mixed models were log-transformed prior to analysis; however, results were back-transformed to the linear scale for clarity of presentation. Statistically significant results and corresponding *p*-values are highlighted in bold.

CI, confidence interval; IM, intermediate metabolizer; NM, normal metabolizer; PM, poor metabolizer; UM, ultrarapid metabolizer.

For participants genotyped for *CYP2C19*, no significant differences in clozapine CD were observed between CYP2C19 genotype-predicted metabolizer phenotypes, either among smokers (PM vs NM, *p* = 0.47; IM vs NM, *p* = 0.09; UM vs NM, *p* = 0.23; Table 3) or nonsmokers (PM vs NM, *p* = 0.12; IM vs NM, *p* = 0.23; UM vs NM, *p* = 0.53; Table 3). Similarly, we did not observe any significant effect of genotype-predicted CYP2D6 phenotypes on clozapine CD, for both smokers (PM vs NM, *p* = 0.09; IM vs NM, *p* = 0.65; IM+ vs NM, *p* = 0.36; UM vs NM, *p* = 0.65; Table 3) and nonsmokers (PM vs NM, *p* = 0.96; IM vs NM, *p* = 0.77; IM+ vs NM, *p* = 0.85; UM vs NM, *p* = 0.85; Table 3).

When examining the impact of *CYP3A4* genotype, we did not observe any significant differences in clozapine CD between carriers and non-carriers of the CYP3A4*22 variant allele, a result that was consistent in both smokers (*p* = 0.28) and non-smokers (*p* = 0.51). However, among nonsmokers, carriers of the CYP3A5*1/*1 genotype exhibited a 37% lower clozapine CD compared to CYP3A5*3/*3 carriers (fold-change: 0.63; 95% CI: 0.42–0.94; *p* = 0.024; Table 3). On the contrary, no significant effect of the *CYP3A5* genotype was observed among smokers (*1/*3 vs *3/*3, *p* = 0.10; *1/*1 vs *3/*3, *p* = 0.57; Table 3).

**Figure 1.**
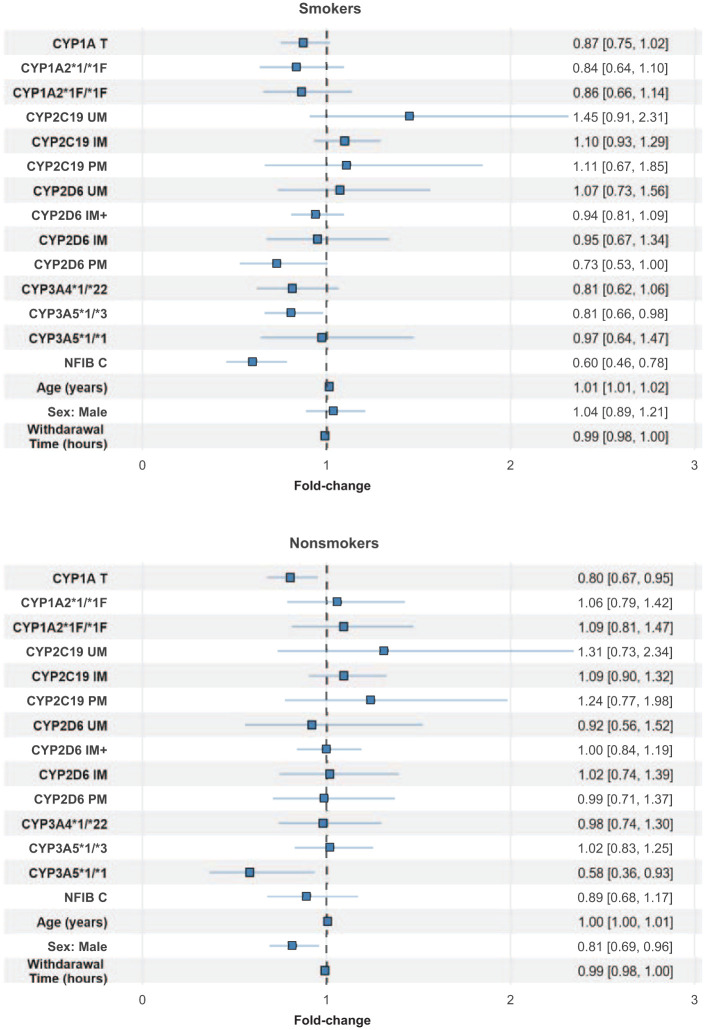
Forest plot showing associations of *CYP-* and *NFIB* genotypes with clozapine dose-adjusted serum concentration (CD) according to smoking status. The mixed-model analyses were performed log-transformed clozapine CD; however, estimated effects were back-transformed into linear scale as fold changes with their corresponding 95% confidence intervals.

We observed that carriers of the *NFIB C* variant were associated with 40% decreased clozapine CD compared to *TT* carriers among smokers (fold-change: 0.60; 95% CI: 0.49–0.74; *p* < 0.001; Table 3), whereas there was no significant effect of the *NFIB C* variant among nonsmokers (*p* = 0.39; Table 3). Finally, when we included all the genetic variants and longitudinal clozapine CDs in a common mixed-model analysis (Figure 1 and Table 4), we observed that, among smokers (*n* = 230 participants with 3300 clozapine samples), clozapine CD was significantly reduced in *NFIB C* variant allele carriers compared *NFIB TT* carriers (fold-change: 0.60; 95% CI: 0.46–0.78; *p* < 0.001). Interestingly, among smokers, but not nonsmokers, CYP2D6 PMs had, with a *p*-value just above the significance threshold, reduced clozapine CD compared to CYP2D6 NMs (fold-change: 0.73; 95% CI: 0.53–1.0; *p* = 0.054). Among nonsmokers (*n* = 185 participants with 2454 clozapine samples), there were significant reductions in clozapine CD in *CYP1A T* variant allele carriers compared to *CYP1A CC* carriers (fold-change: 0.80; 95% CI: 0.67–0.95; *p* = 0.012) and in CYP3A5 *1/*1 participants compared to CYP3A5 *3/*3 carriers (fold-change: 0.58; 95% CI: 0.36–0.93; *p* = 0.025; Figure 1 and Table 4). Furthermore, the combined impact of *CYP1A, CYP3A5*, and/or *NFIB* risk alleles on clozapine CD among smokers and nonsmokers were presented in Table S1. Among smokers, individuals carrying both the *CYP1A T* and *NFIB C* variants exhibited a 50% reduction in clozapine CD compared to noncarriers of these variants (*p* < 0.0001). This effect size was comparable to the cumulative impact of the individual variant genotypes on CD (Table S1). Among nonsmokers, we observed a 58% reduction in clozapine in carriers of *CYP1A T* variant and CYP3A5*1/*1 genotype compared to noncarriers (*p* = 0.080), which was comparable to CD reductions associated with the individual genotypes (Table S1). Due to the unexpected observation of reduced level of clozapine in smoking CYP2D6 PMs, we conducted an additional mixed-model analysis to investigate the impact of comedication with CYP2D6 inhibitors (bupropion, fluoxetine, and paroxetine) on clozapine levels both among smokers and nonsmokers. Our analysis did not show any significant differences in clozapine CD between participants using CYP2D6 inhibitors and CYP2D6*1/*1 genotyped participants who were not taking any medications known to interact with clozapine metabolism, either among smokers (*p* = 0.26; Table S2) or nonsmokers (*p* = 0.70).

**Table 4.** Mixed-model analyses including all genotypes for prediction of dose-adjusted serum concentration (nmol/L/mg/day; CD) of clozapine among smokers and nonsmokers.

Predictor (Fixed effects)	Fold-change (95% CI)	*p* Value
**Smokers**		
*CYP1A TC or TT*	0.87 (0.75, 1.02)	0.081
CYP1A2*1/*1F	0.84 (0.64, 1.1)	0.19
CYP1A2*1F/*1F	0.86 (0.66, 1.14)	0.29
CYP2D6 PM	**0.73 (0.53, 1.0)**	**0.054**
CYP2D6 IM	0.95 (0.67, 1.34)	0.77
CYP2D6 IM+	0.94 (0.81, 1.09)	0.42
CYP2D6 UM	1.07 (0.73, 1.56)	0.72
CYP2C19 PM	1.11 (0.67, 1.85)	0.69
CYP2C19 IM	1.10 (0.93, 1.29)	0.27
CYP2C19 UM	1.45 (0.91, 2.31)	0.12
CYP3A4*1/*22	0.81 (0.62, 1.06)	0.13
CYP3A5*1/*3	0.81 (0.66, 0.98)	0.31
CYP3A5*1/*1	0.97 (0.64, 1.47)	0.90
*NFIB CT or CC*	**0.60 (0.46, 0.78)**	**<0.001**
Age (years)	**1.01 (1.01, 1.02)**	**<0.001**
Sex: Male	1.04 (0.89, 1.21)	0.64
Withdrawal time (hours)	**0.99 (0.98, 1.0)**	**0.002**
**Nonsmokers**		
*CYP1A TC or TT*	**0.80 (0.67, 0.95)**	**0.012**
CYP1A2*1/*1F	1.06 (0.79, 1.42)	0.71
CYP1A2*1F/*1F	1.09 (0.81, 1.47)	0.57
CYP2D6 PM	0.99 (0.71, 1.37)	0.94
CYP2D6 IM	1.02 (0.74, 1.39)	0.92
CYP2D6 IM+	1.0 (0.84, 1.19)	0.99
CYP2D6 UM	0.92 (0.56, 1.52)	0.74
CYP2C19 PM	1.24 (0.77, 1.98)	0.37
CYP2C19 IM	1.09 (0.9, 1.32)	0.37
CYP2C19 UM	1.31 (0.73, 2.34)	0.36
CYP3A4*1/*22	0.98 (0.74, 1.3)	0.88
CYP3A5*1/*3	1.02 (0.83, 1.25)	0.86
CYP3A5*1/*1	**0.58 (0.36, 0.93)**	**0.025**
*NFIB CT or CC*	0.89 (0.68, 1.17)	0.41
Age (years)	**1.0 (1.0, 1.01)**	**0.024**
Sex: Male	**0.81 (0.69, 0.96)**	**0.013**
Withdrawal time (hours)	**0.99 (0.98, 1.0)**	**0.003**

Linear mixed-model analyses were performed with log-transformed dependent variables. Coefficients are converted to linear-scale as fold-changes for presentation of the results. Statistically significant results and corresponding *p*-values are highlighted in bold.

CI, confidence interval; IM, intermediate metabolizer; NM, normal metabolizer; PM, poor metabolizer; UM, ultrarapid metabolizer.

## Discussion

Here, we investigated the impact of pharmacogenetic variability in *CYP-* and *NFIB* genes on clozapine serum levels in patients stratified by smoking habits. The study included 663 participants with longitudinal clozapine TDM profiles with information on comedications that may interact with clozapine metabolism. While the *CYP1A T* variant allele was associated with modest reduction of clozapine CD both among smokers and nonsmokers, the other significant variants were either relevant in smokers or nonsmokers (not both).

In line with previous studies,^24,36^ approximately a 40% reduction of clozapine CD associated with the *NFIB C* variant in smokers. Interestingly, CYP3A5*1/*1 carriers showed a significant reduction of 37% in nonsmokers only. This provides firm evidence that CYP3A5 genotype is important for dose requirements to achieve target concentrations in nonsmokers. The absence of a *CYP3A5* genotype effect in smokers may indicate that clearance is already substantially increased in this group. CYP3A status of the patients has been reported to effect clozapine serum levels and adverse effects.^28,40,50^ CYP3A4*22 variant allele has been shown to reduce CYP3A4 activity and been implemented in pharmacogenetic panels due to some evidence indicating its significant association with metabolism of other CYP3A4 substrates.^51,52^ However, in the current study, we did not observe any significant changes in clozapine CD associated with CYP3A4*22 variant allele carriers. This is in line with previous evidence showing limited value of *CYP3A4* genotyping of known alleles for prediction of CYP3A4 phenotype.^21,28^ In contrast, CYP3A5*1/*1 carriers were significantly associated with significant reduction in clozapine CD among nonsmokers. This is probably due to the limited role of CYP3A in clozapine metabolism under high CYP1A2 activity,^28^ in this case induction with tobacco smoking.

We did not observe any significant effect of CYP1A2*1F variant allele on clozapine serum levels, regardless of smoking habits. Although previous studies reported significant associations of clozapine levels with CYP1A2*1F variant allele,^26,34,53,54^ we could not replicate this finding and our results are in line with the current consensus on clozapine-gene interactions stating current status of *CYP1A2* genotyping do not provide sufficient information warranting dose adjustments.^31,32^ On the other hand, *CYP1A T* and *NFIB C* variant alleles had significant and cumulative effects on clozapine levels among smokers, necessitating increased dosage to achieve target levels compared to patients not carrying these variants. Although *NFIB* silencing was shown to influence the expression of CYP1A2 (but not CYP3A4),^55^ in our previous study, the interaction analysis between *CYP1A* and *NFIB* variants yielded no significant results, thus indicating no evidence of epistasis.^36^ This was perhaps expected as there is a big overlap of participants in the current study and our previous study^36^; however, here, we provide a more robust evidence by applying a more strict criteria with more detailed comedication use and replicating these findings in our models including other relevant *CYP* genotypes and important covariates. To our current knowledge, there is no genetic linkage or biological relationship between the CYP3A5 *1, CYP*1A T*, and/or *NFIB C* alleles; therefore, they represent distinct genetic signals. Accordingly, we observed additive effects of the *CYP1A T* and CYP3A5*1/*1 on clozapine CD among nonsmokers, which further highlights the importance of evaluating the impact of these risk alleles (CYP3A5 *1, CYP*1A T*, and/or *NFIB C*) individually in relation to smoking habits of the patients.

There was no significant association of *CYP2C19* genotype-predicted phenotypes on clozapine levels. Considering the weak previous evidence^25,26,28^ and lack of correlations in the current study, *CYP2C19* genotyping would not provide any clinical utility for individualized dosing of clozapine. An unexpected finding was the association of CYP2D6 metabolizer phenotype, when adjusting for all the other genotypes in the overall mixed-model analysis, with a reduction of clozapine levels in CYP2D6 PMs. *CYP2D6* genotype has been implicated to play a role in clozapine metabolism, as indicated in the FDA package insert, but several studies have not been able to show any effect of *CYP2D6* genotype on clozapine level.^25,28,34,56,57^ In contrast, we observed a reduction of clozapine concentration in CYP2D6 PMs, which does not comply with (i) the fact that these patients inherently lack metabolism, and (ii) the limited involvement of CYP2D6 in clozapine metabolism. Interestingly, no effect on clozapine concentration was present in patients comedicated with potent CYP2D6 inhibitors, regardless of smoking habits, which shows that the reduced CYP2D6 metabolism per se is not the reason for reduced clozapine concentration, but rather linked to a regulatory mechanism associated with being a CYP2D6 PM. This finding aligns with the observation that reduced clozapine levels were only noted in smoking patients, where CYP1A2 expression is induced, and no effect was seen in nonsmoking CYP2D6 PMs. We speculate that the inducibility of other enzymes might be more sensitive due to the absence of a specific enzyme. However, this is only a hypothesis which requires explanation of underlying molecular mechanisms and to be replicated in other populations.

Current treatment guidelines recommend TDM to guide clozapine therapy due to its narrow therapeutic index and the strong relationship between serum concentration and clinical efficacy.^6,12^ For many patients, a serum concentration of 350 ng/mL (1070 nmol/L) is likely required for clinical response.^10^ Although dose titration guided by TDM can be used to achieve target concentrations, early dose adjustments during treatment initiation are critical. Employing advanced prediction algorithms that utilize clinical, environmental, and genetic factors^30^ to accurately estimate clozapine serum levels could facilitate reaching therapeutic clozapine exposure, thereby shortening the time to clinical response in patients with TRS, who often suffer from a more severe disease course. Accordingly, findings in the current study suggest that pre-emptive genotyping of *CYP1A*, *NFIB*, and *CYP3A5*, in combination with information on smoking habits, should be utilized for personalized dosing of clozapine. So far, we consider the evidence to be insufficient for recommending higher clozapine dosing in CYP2D6 PMs for smokers.

### Limitations

Our study has several limitations inherent to naturalistic/observational designs. It was not possible to account for all potential confounding variables, such as ancestry, renal/hepatic function, inflammatory status, and body weight, each of which may influence clozapine pharmacokinetics. It is possible that not all drugs interacting drugs may have been reported on the TDM requisition forms. However, usually important drug interactions with clozapine are listed, and we have reprocessed the last measured clozapine samples for a large part of our population to have full coverage of interacting drugs. Participants who were on any relevant comedications were excluded from the study. Furthermore, the study includes a large number of participants and repeated clozapine serum concentration measurements collected over an extended period, combined with comprehensive data on key confounding factors, particularly smoking status. These strengths likely outweigh the noted limitations and improve the precision of our estimates regarding genotype-associated clozapine levels.

## Conclusion

In conclusion, *CYP1A T*, *NFIB C*, and CYP3A5*1 alleles are associated with significant reductions in clozapine serum levels. The inclusion of genotype information of these genes, alongside smoking habits, would improve algorithms for precision dosing of clozapine. The current findings warrant further studies to reveal the underlying molecular mechanisms behind the reduced clozapine serum levels associated with CYP2D6 PMs.

## Supplemental Material

sj-docx-1-tpp-10.1177_20451253251377183 – Supplemental material for Impact of CYP1A, CYP2C19, CYP2D6, CYP3A4, CYP3A5, and NFIB genotypes on clozapine serum concentration in smokers and nonsmokers Supplemental material, sj-docx-1-tpp-10.1177_20451253251377183 for Impact of CYP1A, CYP2C19, CYP2D6, CYP3A4, CYP3A5, and NFIB genotypes on clozapine serum concentration in smokers and nonsmokers by Hasan Çağın Lenk, Line Skute Bråten, Ole A. Andreassen and Espen Molden in Therapeutic Advances in Psychopharmacology

sj-docx-2-tpp-10.1177_20451253251377183 – Supplemental material for Impact of CYP1A, CYP2C19, CYP2D6, CYP3A4, CYP3A5, and NFIB genotypes on clozapine serum concentration in smokers and nonsmokers Supplemental material, sj-docx-2-tpp-10.1177_20451253251377183 for Impact of CYP1A, CYP2C19, CYP2D6, CYP3A4, CYP3A5, and NFIB genotypes on clozapine serum concentration in smokers and nonsmokers by Hasan Çağın Lenk, Line Skute Bråten, Ole A. Andreassen and Espen Molden in Therapeutic Advances in Psychopharmacology

## Declarations

**Ethics approval and consent to participate:** This study received ethical approval from the Regional Committee for Medical and Health Research Ethics (ref #9393) and the Hospital Investigational Review Board (IRB). This is an IRB-approved retrospective study, all patient information was de-identified, and patient consent was not required.

**Consent for publication:** Not applicable.

**Availability of data and materials:** The data that support the findings of this study are available from the corresponding author upon reasonable request. The data are not publicly available due to privacy restrictions.
