# Supplementary material for: Impact of CYP1A, CYP2C19, CYP2D6, CYP3A4, CYP3A5, and NFIB genotypes on clozapine serum concentration in smokers and nonsmokers
Source: Ther Adv Psychopharmacol. 2025 Oct 4;15:20451253251377183. doi: 10.1177/20451253251377183 (PMC12496473; doi:10.1177/20451253251377183)
Supplement: sj-docx-2-tpp-10.1177_20451253251377183 – Supplemental material for Impact of CYP1A, CYP2C19, CYP2D6, CYP3A4, CYP3A5, and NFIB genotypes on clozapine serum concentration in smokers and nonsmokers [file sj-docx-2-tpp-10.1177_20451253251377183.docx]

**Impacts of CYP1A, CYP2C19, CYP2D6, CYP3A4, CYP3A5, and NFIB genotypes on clozapine serum concentration in smokers and nonsmokers**

**Supplementary Materials**

**Table S1** Estimated means of dose-adjusted serum concentrations (nmol/L/mg/day; CD) of clozapine according to different *CYP1A, CYP3A5,* and *NFIB* combined genotypes in smokers and nonsmokers.

| **Genotype** | | **n, subjects (samples)** | **Mean (95%CI)** | **Fold-change (95%CI)** | **p-value** |
| --- | --- | --- | --- | --- | --- |
| **Smokers** | |  |  |  |  |
|  | *CYP1A C/C* – *NFIB* *T/T* (**ref**) | 166 (2698) | 2.92 (2.69, 3.17) | — | — |
|  | *CYP1A T* carriers – *NFIB T/T* | 114 (1568) | 2.57 (2.34, 2.83) | 0.88 (0.78, 1.0) | 0.042 |
|  | *CYP1A* *C/C* – *NFIB C* carriers | 14 (97) | 1.97 (1.48, 2.62) | 0.68 (0.50, 0.91) | 0.009 |
|  | *CYP1A T* carriers – *NFIB C* carriers | 13 (257) | 1.44 (1.09, 1.91) | 0.50 (0.37, 0.66) | <0.0001 |
| **Nonsmokers** | |  |  |  |  |
|  | *CYP1A C/C* – *CYP3A5*3/*3* (**ref**) | 106 (1679) | 4.63 (4.20, 5.11) | — | — |
|  | *CYP1A T* carriers – *CYP3A5*3/*3* | 78 (1167) | 3.83 (3.42, 4.29) | 0.83 (0.71, 0.96) | 0.014 |
|  | *CYP1A C/C* – *CYP3A5*1/*1* | 6 (26) | 2.85 (1.85, 4.39) | 0.62 (0.40, 0.96) | 0.032 |
|  | *CYP1A T* carriers – *CYP3A5*1/*1* | 1 (10) | 1.93 (0.73, 5.11) | 0.42 (0.16, 1.11) | 0.080 |

Multivariate linear mixed-effects models were used to estimate the impact of clozapine CD, separately among smokers and nonsmokers. All models included age, sex, and withdrawal time as covariates. Outcome variables in the mixed models were log-transformed prior to analysis; however, results were back-transformed to the linear scale for clarity of presentation.

CI, Confidence interval

|  |  |
| --- | --- |

| **Table S2** Estimated means and comparisons of dose-adjusted serum concentration (CD) of clozapine between *CYP2D6*1/*1* carriers and participants comedicated with CYP2D6 inhibitors | | | | | |
| --- | --- | --- | --- | --- | --- |
| Subjects | | n, subject (measurement) | Estimated Mean (95%CI) | Fold change (95% CI) | *p* Value |
| **Smokers** | | | | | |
|  | *CYP2D6*1/*1* | 124 (1481) | 2.80 (2.52, 3.12) | —— | —— |
|  | Comedicated with CYP2D6 Inhibitors | 23 (95) | 3.29 (2.55, 4.24) | 1.17 (0.89, 1.54) | 0.26 |
| **Nonsmokers** | | | | | |
|  | *CYP2D6*1/*1* | 100 (954) | 4.30 (3.86, 4.79) | —— | —— |
|  | Comedicated with CYP2D6 Inhibitors | 13 (103) | 4.58 (3.4, 6.17) | 1.07 (0.78, 1.46) | 0.70 |
| Multivariate linear mixed-effects models were used to estimate the impact of clozapine CD, separately among smokers and nonsmokers. All models included age, sex, and withdrawal time as covariates. Outcome variables in the mixed models were log-transformed prior to analysis; however, results were back-transformed to the linear scale for clarity of presentation. | | | | | |
| *CYP2D6*1/*1* carriers were not comedicated with interacting drugs, while participants comedicated with CYP2D6 inhibitors were not genotype-predicted poor metabolizers | | | | | |
| CYP2D6 Inhibitors: Bupropion, Fluoxetine, Paroxetine | | | | | |
| CI, confidence interval | | | | | |
